# Object words modulate the activity of the mirror neuron system during action imitation

**DOI:** 10.1002/brb3.840

**Published:** 2017-09-26

**Authors:** Haiyan Wu, Honghong Tang, Yue Ge, Suyong Yang, Xiaoqin Mai, Yue‐Jia Luo, Chao Liu

**Affiliations:** ^1^ CAS Key Laboratory of Behavioral Science Beijing China; ^2^ Department of Psychology University of Chinese Academy of Sciences Beijing China; ^3^ State Key Laboratory of Cognitive Neuroscience and Learning & IDG/McGovern Institute for Brain Research Beijing Normal University Beijing China; ^4^ School of Economics and Business Administration Beijing Normal University Beijing China; ^5^ Beijing Institution of Biomedicine Beijing China; ^6^ Key Laboratory of Exercise and Health Sciences of Ministry of Education Shanghai University of Sport Shanghai China; ^7^ Department of Psychology Renmin University of China Beijing China; ^8^ Institute of Affective and Social Neuroscience Shenzhen University Shenzhen Guangdong China; ^9^ Center for Collaboration and Innovation in Brain and Learning Sciences Beijing Normal University Beijing China; ^10^ Beijing Key Laboratory of Brain Imaging and Connectomics Beijing Normal University Beijing China

**Keywords:** action words, fMRI, imitation, mirror neuron, objects

## Abstract

**Background:**

Although research has demonstrated that the mirror neuron system (MNS) plays a crucial role in both action imitation and action‐related semantic processing, whether action‐related words can inversely modulate the MNS activity remains unclear.

**Methods:**

Here, three types of task‐irrelevant words (body parts, verbs, and manufactured objects) were presented to examine the modulation effect of these words on the MNS activity during action observation and imitation. Twenty‐two participants were recruited for the fMRI scanning and remaining data from 19 subjects were reported here.

**Results:**

Brain activity results showed that word types elicited different modulation effects over nodes of the MNS (i.e., the right inferior frontal gyrus, premotor cortex, inferior parietal lobule, and STS), especially during the imitation stage. Compared with other word conditions, action imitation following manufactured objects words induced stronger activation in these brain regions during the imitation stage. These results were consistent in both task‐dependent and ‐independent ROI analysis.

**Conclusion:**

Our findings thus provide evidence for the unique effect of object words on the MNS during imitation of action, which may also confirm the key role of goal inference in action imitation.

## 1 INTRODUCTION

Neuroimaging studies have provided abundant and strong evidence for the role of the mirror neuron system (MNS) in action observation and imitation (Iacoboni & Dapretto, 2006; Iacoboni et al., 1999; Molenberghs, Brander, Mattingley, & Cunnington, 2010; Molnar‐Szakacs, Kaplan, Greenfield, & Iacoboni, 2006; Montgomery & Haxby, 2008; Rizzolatti, Cattaneo, Fabbri‐Destro, & Rozzi, 2014; Rizzolatti & Craighero, 2004; Rizzolatti, Fogassi, & Gallese, 2001; Rizzolatti & Sinigaglia, 2010). These data clearly show that a frontoparietal mirror neuron system underlying imitation coincides with that which is active during action observation. Experimental data also suggest that different parts of the mirror neuron respond to different kinds of actions (Bonini, Maranesi, Livi, Fogassi, & Rizzolatti, 2014; Caggiano, Fogassi, Rizzolatti, Thier, & Casile, 2009; Caggiano et al., 2007; Maeda, Ishida, Nakajima, Inase, & Murata, 2015). For instance, the frontal mirror region (inferior frontal gyrus) has been demonstrated to be activated when observing the goals of hand–object interactions (Johnson‐Frey et al., 2003). The intention understanding function of the inferior frontal gyrus is confirmed in an fMRI study investigating the brain activity when observing actions embedded or not embedded in contexts (Iacoboni et al., 2005a). Nevertheless, a study in monkeys suggests that the discharge of the inferior parietal lobule (IPL) mirror neurons is influenced by the prediction about the final goal of the neuronal discharges (Fogassi et al., 2005). Therefore, the functional and cognitive roles of the frontal and parietal mirror neuron regions require further investigation.

Although with controversy (Mikulan, Reynaldo, & Ibanez, 2014), the mirror neuron system has been suggested to play an important role in the evolution of human language (Rizzolatti & Arbib, 1998). A growing body of literature has shown that action‐related words, especially verbs and man‐made artifact nouns, share neural substrates in the human somatosensory cortex, which are linked to the MNS (Buccino et al., 2005; Hauk, Johnsrude, & Pulvermuller, 2004; Moreno, de Vega, & Leon, 2013; Pulvermüller, 2005; Tettamanti et al., 2005; de Zubicaray, Postle, McMahon, Meredith, & Ashton, 2010). Evidence also shows that the processing of action words affects motor preparation and execution (Boulenger et al., 2006; Nazir et al., 2008). One representative work from Boulenger et al. (2006) showed the acceleration of action execution following action verb processing in the early time window, indicating a direct link between language processes and the overt motor behavior. Furthermore, an EEG study revealed that subliminally presented action words reduced the readiness potential and interfered with subsequent motor action (Boulenger et al., 2008). Despite evidence that language meaning is embodied in the motor system, whether and how language per se, such as single words without context can modulate the motor system during action observation or execution, especially over the MNS, is still unexplored, even though such a modulation effect would be strongly predicted on the basis of the overlapped brain areas between language and the MNS.

In everyday life, motor imitation can be influenced by providing verbal instructions but also disrupted by task‐irrelevant single words. The fMRI studies have investigated the underlying brain mechanisms by setting up the link between verbal cues and motor responses, and demonstrated that the inferior frontal junction (IFJ) guides modality‐specific areas needed to perform the upcoming task and the dorsal fronto‐median cortex (dFMC) is associated with motor inhibition (Hartstra, Waszak, & Brass, 2012; Kühn, Haggard, & Brass, 2009). Behaviorally, the connection between the MNS and speech has been demonstrated by a study showing that articulation interferes with the imitation task (Kuhn & Brass, 2008). Specifically, with a dual task paradigm (say “SALAMANDER!” in an imitation task), the imitation reaction time was more prolonged than a control task without articulation, which confirmed that the speaking of a task‐irrelevant single word interfered with the imitation task and indicated a possible functional overlap between speech and imitation. However, the task‐irrelevant words could be classified into different types based on their usage in real life, such as subject words (i.e., denoting the subject of the action), verbs (i.e., description of the action per se), and object words (i.e., the goal or the target of the action). According to the link between language and the motor cortex proposed by Pulvermüller (2005), verbs have a particular overlap with the motor and premotor cortex, which also may indicate a functional overlap with the MNS. Therefore, we hypothesized that different from subject and object words, the verbs may show interference with the imitation task and modulate brain activity in the MNS. While viewing graspable objects can activate the F5 area in macaque monkeys (Rizzolatti et al., 1988) and the premotor‐parietal cortex in the human brain (Creem‐Regehr & Lee, 2005), and the MNS was consistently found to be involved in goal understanding (Bach, Peelen, & Tipper, 2010; Thioux & Keysers, 2015), single object words might interfere with the imitation task, showing greater activity in the MNS.

Based on these hypotheses, and considering action‐related words, action observation and execution are closely linked to the sensory motor cortex, one principal interest of this study was to examine the modulation effect of different types of task‐irrelevant words (i.e., words that are not the same as or connected with the action itself) on the sensory motor cortex during action observation and imitation. We investigated the way that *different task‐irrelevant* words (e.g., subject, verb, and action object words) modulate mirror neuron activation during action observation and execution, through presenting single words before an action observation–imitation task. We hypothesized that by presenting different types of words before action observation and imitation, those task‐irrelevant words may interfere with action imitation, which modulates brain activity over action observation and the action imitation motor system, especially in the MNS. Specifically, when the words are irrelevant to the actions in observation and imitation, the MNS might be more active compared to control condition (i.e., the checkerboard), since the brain needs to resist interference between words and its relevant actions.

Further, different types of words might have different interference effect. From the shared brain function perspective, body parts (i.e., subject words) and action words (i.e., verbs) have been shown to activate action observation areas (Buccino et al., 2001, 2005; Hauk et al., 2004). While for object words, a large range of empirical data has provided evidence for the intention or goal representations over the MNS (Binkofski & Buccino, 2006; A. F. D. Hamilton & Grafton, 2006; Iacoboni et al., 2005a; Jarvelainen, Schurmann, & Hari, 2004; Muthukumaraswamy, Johnson, & McNair, 2004; Ocampo & Kritikos, 2011; Ogawa & Inui, 2012). Since object words induce overlapping neural network over the MNS with action imitation, we therefore expected that compared to subject words and verbs, object words would have stronger interference effects on the imitation task.

## 2 MATERIALS AND METHODS

### 2.1 Participants

Twenty‐two participants were recruited from Beijing Normal University and three participants were excluded in the final analysis due to excessive head motion. We thus only reported the relevant information from the remaining 19 participants (10 men, mean age ± *SD* = 24.4 ± 3.0 years; 9 women, mean age ± *SD* = 24.8 ± 3.7 years). All participants were right‐handed and native speakers of Chinese. They were screened medically to rule out any history of neurological or psychiatric disorders, head trauma, and other serious medical conditions. Participants provided written informed consent and the procedure of the experiment was approved by the institutional review board (IRB) of Beijing Normal University.

### 2.2 Materials

**Figure 1:**
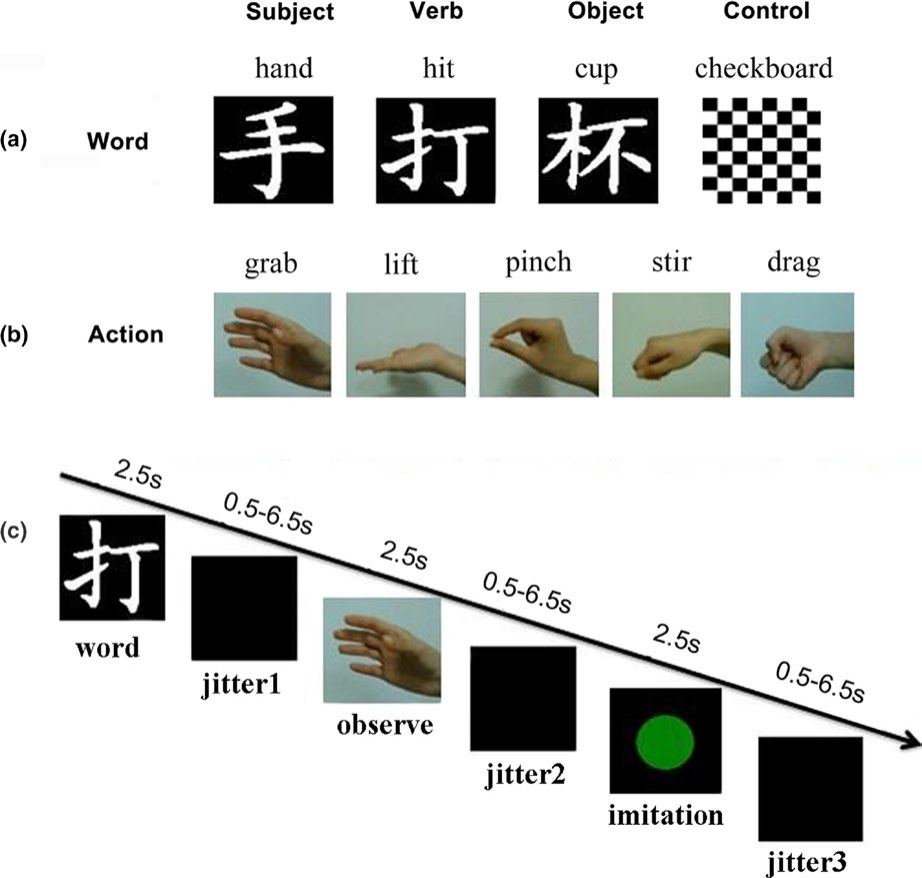
Sample stimuli from the experimental stimuli set and the procedure of each trial: Each trial included three stimuli stages that were displayed for 2500 ms, spaced by three interstage intervals which varied between 500–6500 ms. Participants were asked to read the word silently during the word stage, to view the action video clips in the action observation stage, and to imitate the action when the green dot was presented in the action imitation stage. Duration of the events: Word = 2.5 s; Jitter 1 = 0.5–6.5 s; Observation = 2.5 s; Jitter 2 = 0.5–6.5 s; imitation=2.5 s, Jitter 3 = 0.5–6.5 s

Three types of action‐related Chinese words (subject, verb, and object) were not significantly different in lexical frequency. Specifically, the analysis of variance (ANOVA) on word frequency found no significant word type effect, *F*_2,12_ = 0.128, *p *=* *.88, and the t‐tests failed to find significant differences between any two word types, *ps *> 0.66. The subject words consisted of five body part words related to the hand (e.g., HAND, WIST etc.). Five hand‐related action verbs (e.g., HIT) and five hand action‐related object words (e.g., CUP) were used as verb stimuli and object stimuli, respectively. Additionally, five checkerboards were used as the stimuli in the control condition. Five video clips of hand actions (e.g., GRAB) which were cut with same duration (2s), were used as the imitation target (see Figure 1). All of the word stimuli (including subjects, actions/verbs, and objects) used in the experiment are listed in Table 1. As the table indicated, the verbs are not corresponding to the action clips. Furthermore, the presentation order was pseudo‐random so that the action in the video was always not compatible with the objects.

**Table 1:**
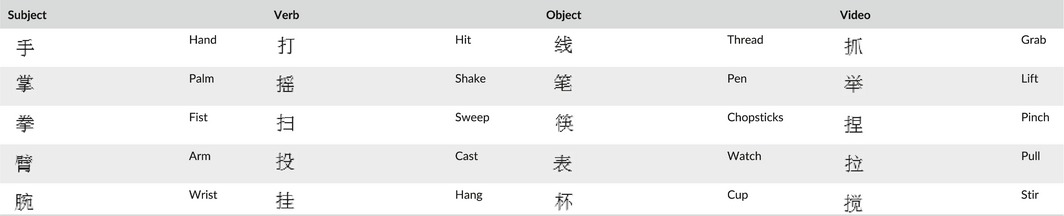
Word materials and the video action in this study

### 2.3 Procedure

Each trial consisted of three stages (word, observation, and imitation) that lasted 2500 ms each, separated by three intervals that varied from 500 to 6500 ms (Figure 1). Two hundred trials were classified into four conditions based on the type of stimuli in the word stage, which included 50 words in each of the three word types and 50 pictures of checkerboard. Participants were instructed to either read the words (i.e., subject, verb, and object words) silently or keep attending to the checkerboard. In the observation stage, participants were asked to view the video clip of hand action and prepare to imitate the action. Finally, participants needed to imitate the action in video clips with their right hand just one time when a green dot appeared as a cue and then kept still after the dot disappeared (Figure 1). To ensure all participants performed the imitation task as instructed in each trial, an experimenter standing outside the scanner observed and recorded the performance of each participant with a 5‐point rating scale(“1” represents bad performance of action imitation, “5” represents very good performance). The recording indicated that all participants’ imitation were above 4 points, which suggested that actions were well imitated in the scanner.

Subsequent to the main task, all participants completed an action‐naming task on all five movie clips (i.e., “what is the action in the video clip?”) outside the scanner to verify all words were different from actions in the movie clips even both of them were hand action related. For instance, the action in the movie clip was “pull,” while the presented word was “hit.” No participant reported the name of actions as the same in the word stage in the main task.

### 2.4 MRI data acquisition and analysis

All imaging data were acquired on a 3T Siemens scanner with an upgrade for echo‐planar imaging (EPI). For each participant, a high‐resolution T1 structural image (spin‐echo, TR = 4,000 ms, TE 54 ms, 128 by 128, 26 slices, voxel size 1 × 1 × 1 mm^3^ ‐mm spacing) was scanned to allow subsequent activation localization and spatial normalization. Four functional EPI runs (TR = 1500 ms, TE = 28 ms; acquisition matrix = 64 × 64; flip angle 75°; in‐plane resolution = 3.1 × 3.1 mm^2^; and field of view = 200 × 200 mm) were scanned, with each run lasting approximately 6 min. Each functional scan consisted of 28 slices covering the whole brain (slice thickness was 3 mm). The first three scans were excluded in the analyses due to the expected initial signal instability in the functional scans. Image processing was carried out with SPM5 (Wellcome Department of Imaging Neuroscience, London, UK) implemented in MATLAB 7.1 (Mathworks Inc. Sherborn, MA, RRID: SCR_001622). Scans were first preprocessed for slice‐timing, realignment, normalization (to MNI space), and smoothing (8 × 8 ×  8 mm, Gaussian spatial filter). The resulting images had a voxel size of 3.13 × 3.13 × 4.8 mm^3^.

Event‐related activity for each voxel, condition, and subject was modeled using a canonical hemodynamic response function plus temporal and dispersion derivatives. Regressors of interest modeling the four experimental conditions (subject, verb, object, and checkerboard) in three stages (word, observation, and imitation) were respectively convolved with a canonical hemodynamic response function (hrf), resulting in 12 statistical parametric maps of the t‐statistic in the first‐level analysis (uncorrected voxel‐wise of *p *<* *.001, *k *>* *10 voxels).

First, we analyzed the overall activation in three stages, respectively, and overlapped activations in the observation and imitation stage. To investigate the effect of word type, analyses were conducted to determine changes related to word type (subject, verb, object, and checkerboard) based on one‐way repeated measures ANOVA with the within‐subjects factor of word type (uncorrected voxel‐wise of *p *<* *.001 and cluster threshold of *p *<* *.01 to protect against false positives, *k *>* *10 voxels). We only found a significant main effect of word type, mostly in the occipital cortex, at the word stage and did not find any significant word type effect at the observation stage. However, we found a significant main effect of word type at the imitation stage over several frontoparietal brain regions. Therefore, ROI analyses were conducted using the tool SPM5 (RRID:SCR_007037, http://www.fil.ion.ucl.ac.uk/spm/software/spm5/) and MarsBar (RRID: SCR_009605, http://marsbar.sourceforge.net/) for 4‐peak activation clusters (sphere 10 mm) from the map of word type main effect at the imitation stage. The percent signal change data were analyzed in SPSS17.0 (RRID:SCR_002865) in a 4 (word type:subject, verb, object, and checkerboard) ×3 (stage: word, observation, and imitation) ANOVA first, and then with a 4‐level one‐way ANOVAs focusing the word effect in the imitation stage. To exclude the possible difference in the control condition (checkerboard) versus word conditions, and in word stage versus action stage, we conducted a 3 (word type: subject, verb, and object) × 2 (stage: observation and imitation) ANOVA.

In addition, we performed an independent ROI analysis, similar to Chong, Cunnington, Williams, Kanwisher, and Mattingley (2008), Chong, Williams, Cunnington, and Mattingley (2008), over putative bilateral mirror areas by constructing spheres of 10 mm radius with the coordinates. Specially, the coordinates were averaged from previous studies of action observation, execution, and/or imitation. Six MNI coordinates (left vPM: −52, 5, 24; right vPM: 52, 11, 20; left aIPS: −38, −46, 49; right aIPS: 32, −52, 54; left STS: −52, −46 4; right STS: 60, −44, 12) were transformed from Talairach coordinates as in the studies of Chong, Cunnington, et al. (2008), Chong, Williams, et al. (2008), and Dinstein, Hasson, Rubin, and Heeger (2007). The extracted percent signal change data of each ROI was put into a Word type (subject, verb, object, and checkerboard) × Stage (word, observation, and imitation) ANOVA and then a 4‐level one‐way ANOVA on word type at the imitation stage as in the previous ROI analysis. The Greenhouse Geisser epsilon correction was implemented to adjust the degrees of freedom of the F‐ratios.

## 3 RESULTS

### 3.1 Activation in each stage

**Figure 2:**
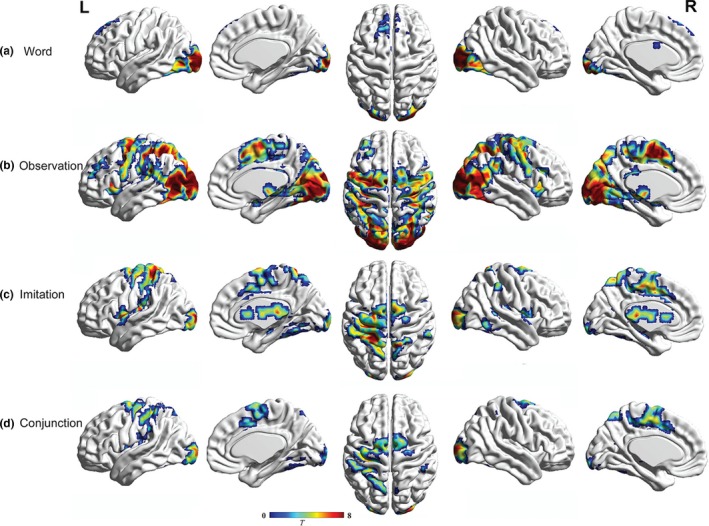
Overall activation in three stages and the overlapping brain regions for action observation and imitation. (a) Activation for word stage, (b) Activation in the stage of action observation, and (c) Activation for imitation stage. The overlapping activation between (b) and (c) are shown in the conjunction analysis (d). Images are shown with a statistical threshold of voxel‐wise uncorrected *p *<* *.001. Coordinates and statistics are provided in Table 2

Figure 2 and Table 2 display the brain areas showing significant activation for the word, observation, and imitation stages. The word reading stage activated the bilateral middle occipital gyrus (BA18) and left cingulate gyrus (BA24). At the observation stage, activation was observed in the middle frontal gyrus (BA6 and BA46), inferior frontal gyrus (BA9), precentral gyrus (BA4), inferior parietal lobule (BA40), cuneus, and bilateral middle occipital gyrus (BA18). For the imitation stage, there was a significant activation in brain areas known to be activated during hand motor imitation (Gatti et al., 2017; Koski et al., 2002; Leslie, Johnson‐Frey, & Grafton, 2004), such as the precentral gyrus (BA6 and BA44), postcentral gyrus (BA3), inferior parietal lobe (BA40), and medial frontal gyrus (BA6). Overall, action observation activated more vision‐related brain areas (occipital lobe), while action imitation activated more premotor areas (i.e., precentral gyrus and postcentral gyrus).

**Table 2:** Coordinates and statistics for activation peaks produced during three phases and the main effect of word type during imitation phase

	BA	x	y	z	*t*(18)	*Z*
Words						
Middle occipital gyrus						
L	18	−25	−94	0	14.12	6.62
R	18	28	−97	0	16.54	7.00
Superior frontal gyrus						
L	6	−9	34	62	6.69	4.68
Medial frontal gyrus						
L	8	−6	50	53	5.19	4.01
Middle frontal gyrus						
L	6	−28	19	62	4.53	3.65
Observation						
Middle occipital gyrus						
L	18	−16	−100	10	18.79	7.3
R	19	−28	−94	14	15.19	6.8
Cuneus						
R	18	16	−97	14	16.36	6.98
Precuneus						
L	7	−25	−69	34	10.47	5.87
	7	28	−69	34	11.27	6.06
Middle frontal gyrus						
L	6	−28	−9	58	12.31	6.28
Medial frontal gyrus						
L	6	−6	−3	53	10.5	5.88
Inferior parietal lobe						
L	40	−31	−41	58	10.36	5.84
Imitation						
Cuneus						
L	18	−19	−103	0	8.7	5.38
R	18	19	−103	5	8.9	5.45
Precentral gyrus						
L	4	−28	−28	58	8.18	5.22
R	6	44	3	10	5.65	4.23
Postcentral gyrus						
L	5	−22	−44	67	11.14	6.03
R	7	13	−53	72	10.17	5.79
Inferior parietal lobe						
R	40	59	−38	48	5.24	4.03
R	40	50	−41	58	4.14	3.43
Superior frontal gyrus						
L	6	−13	−9	77	7.02	4.81
R	6	13	0	77	7.15	4.86
Main effect of word type						
Precentral gyrus						
L	6	−47	−3	53	8.46	3.81
R	44	50	16	10	8.17	3.73
Postcentral gyrus						
L	3	−50	−13	53	6.44	3.22
R	3	47	−22	48	11.29	4.48
Inferior frontal gyrus						
R	45	56	19	19	7.51	3.55
R	45	63	13	24	6.56	3.26
Inferior parietal lobe						
L	40	−50	−59	43	7.9	3.66
L	40	−41	−56	43	6.59	3.27

Regarding the conjunction analysis of brain activity during observation and imitation stages, we observed overlapped activity in the middle occipital gyrus (BA18), left precentral gyrus (BA4), left inferior parietal lobule (BA40), and left superior frontal gyrus (BA6) in the stage of observation and imitation. These brain regions coincide with the classic MNS in the frontal and parietal lobes (Filimon, Rieth, Sereno, & Cottrell, 2015; Rizzolatti & Sinigaglia, 2010), which confirmed that the MNS was involved in the imitation task (see Figure 2d).

### 3.2 The word type effect in different stages

**Figure 3:**
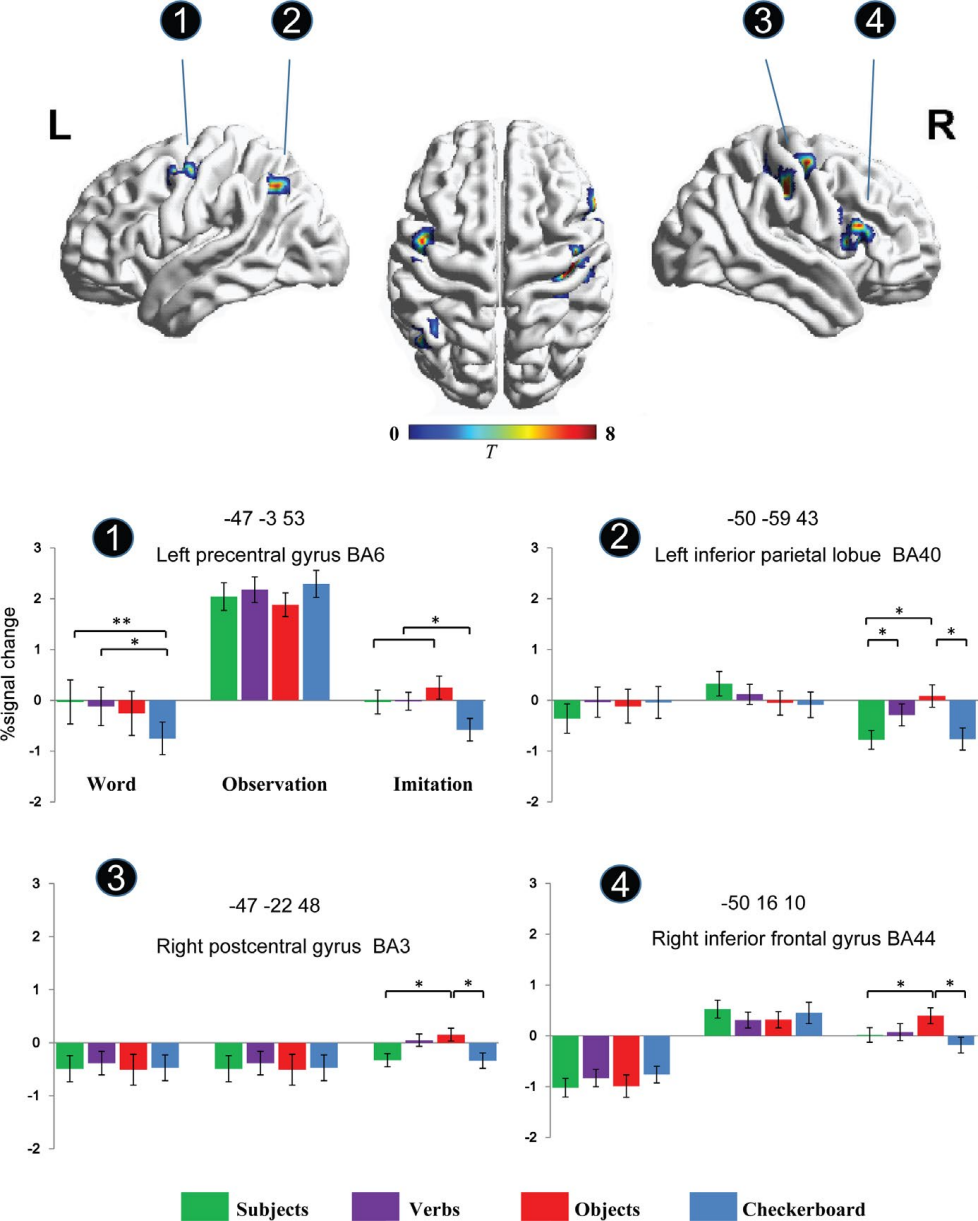
Mean parameter estimates in the four ROIs identified from the ANOVA analyses. The object word condition induced stronger activation in the left IPL, right IFG, and right PostG. The word effect was significant in the left PrecG. * *p *<* *.05, ** *p *<* *.01. Error bars are standard error of the mean

We did not find any significant word effect in the observation stage. However, the 4‐level one‐way ANOVA in the imitation stage revealed the significant main effect of word type in four putative mirror neuron brain regions (left precentral gyrus [PrecG], left inferior parietal lobule [IPL], right postcentral gyrus [PostG], and right inferior frontal gyrus [IFG)]). Activities in these ROIs at the three stages are shown in Figure 3. First, the Word type×Stage ANOVA revealed a significant stage effect in the left precentral gyrus (*F*_2,36_ = 23.50, *p *<* *.001), right postcentral gyrus (*F*_2,36_ = 13.50, *p *<* *.001), and right IFG (*F*_2,36_ = 22.45, *p *<* *.001), indicating the relative strongest activity in the observation stage. Furthermore, the Word×Stage interaction effect was significant for all ROIs, *F*s _(6, 108)_ > 2.77, *p*s < .05, showing a word type modulation effect in the imitation stage. To confirm this result, 4‐level one‐way ANOVAs were performed and the significant effects were reported as below (see Figure 3).

The left precentral gyrus (−47, −3, 53) was the only area that showed the word type effect in the word (*F*_2,34_ = 6.18, *p *<* *.01) and imitation (*F*_3,54_ = 10.310, *p *<* *.001) stages. Subject words and verbs showed stronger activation than control stimuli (checkerboard) in the word reading stage (*ps* < .05), and the control condition had less activation than the other three word conditions (*ps* < .05) in the imitation stage. The language enhancement effect on both the word reading and imitation stages in the left precentral gyrus may have been due to that the semantic processing of hand action‐related words automatically activates the hand action area (Hauk et al., 2004; Postle, McMahon, Ashton, Meredith, & de Zubicaray, 2008; Pulvermüller, 2005; Wu et al., 2013).

In the left IPL (−50, −59, 43), right postcentral gyrus (PostG) (47, −22, 48) and right IFG (50, 16, 10), the significant main effect of word type in the imitation stage (IPL: *F*_3,54_ = 6.133, *p *<* *.01; PostG: *F*_3,54_ = 11.361, *p *<* *.01; IFG: *F*_3,54_ = 6.278, *p *<* *.01) indicated that the object word condition (IPL: *M* = 0.08, SE = 0.22; PostG: *M* = 0.15, SE = 0.12; IFG: *M* = 0.39, SE = 0.16) had a stronger signal change than the subject word (IPL: *M* = −0.78, SE = 0.19; PostG: *M* = −0.33, SE = 0.12; IFG: *M* = 0.02, SE = 0.14) and control condition (IPL: *M* = −0.76, SE = 0.22, *ps* < .05; PostG: *M* = −0.34, SE = 0.14, *ps *< .001; IFG: *M* = −0.18, SE = 0.15, *ps *< .05). Both object words and verbs evoked more activation in IPL than subject words and the checkerboard did, indicating that the mirror neuron system in the bilateral parietal lobe may reflect action representation in the brain (Iacoboni & Dapretto, 2006). Object words induced stronger activation in the right precentral gyrus/inferior frontal gyrus (BA44) than subject words and the checkerboard did, indicating that the mirror neuron system in the right ventral frontal lobe is more sensitive to the pre‐presented object word. Moreover, the verb condition also elicited stronger activity in the right PostG than the subject word and the control condition did (*ps *< .05).

Overall, Figure 3 shows the percent of signal change in each of the ROIs for the four word types in the three stages. As seen, all ROIs responded strongly under the object word condition in the imitation stage. However, no word type modulation effect was found in the observation stage in any of these regions.

To exclude the possibility that the main effect of “word” might be primarily driven by the difference between words and the checkerboard condition, we also performed a 3 (word:subject, verb, and object) × 2 (stage: observation and imitation) ANOVA analysis, and a significant interaction effect was found in the right postcentral gyrus (38, −28, 48), left inferior parietal lobule (−47, −59, 43), and left caudate head (−3, −44, 38) (see Table S1 and Figure S1). The ROI analysis (see also Figure S1) in these brain regions also indicated the highest activity for the object word condition in the imitation stage, which is consistent with the prior results.

### 3.3 Independent ROI analyses

**Figure 4:**
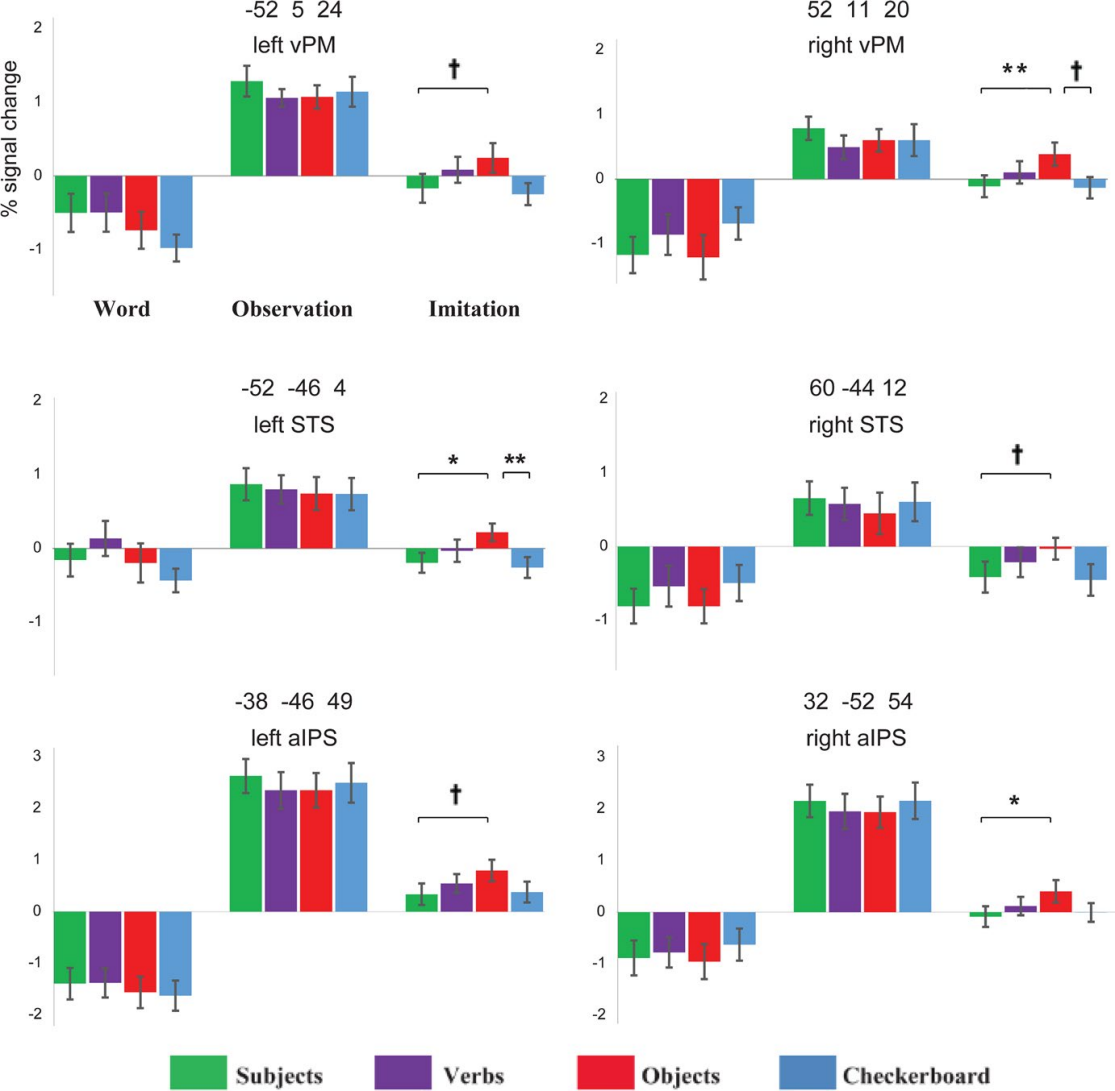
Mean parameter estimates in the six independent ROIs. The object word condition induced the strongest activity over the bilateral vPM, aIPS, and STS only in the imitation stage. The Word × Stage effect was significant in the vPM, aIPS, and STS, indicating that the word effect occured in the imitation stage and object words induced strongest activity than other words and checkerboard. Error bars are standard error of the mean. † *p* < .1, * *p* < .05 ** *p* < .01

To confirm the robust object effect in the imitation stage over MNS nodes, we also conducted ROI analyses over putative bilateral mirror neuron areas (vPM: ventral premotor cortex, aIPS: anterior intraparietal sulcus, and STS: superior temporal sulcus) implicated in previous studies of action observation, execution, and/or imitation. The ROI analysis over these regions showed a stage effect and replicated the robust object word effect in imitation stage. Specifically, the main effect of stage showed the highest activity in the observation stage relative to the imitation stage, *p*s < .05. The stronger activity in the observation stage and relatively weak activity in the imitation stage is somewhat analogous to the fMRI adaption effect or repetition suppression effect on the repeated presentation of specific stimulus (De Lucia et al., 2010; Press et al., 2012; Vuilleumier, Schwartz, Duhoux, Dolan, & Driver, 2005); that is, action observation increased the activity in motor preparation areas during the initial observation period, whereas the execution of the same action evoked neural suppression over the motor areas (Dinstein et al., 2007; Kable & Chatterjee, 2006; Krams, Rushworth, Deiber, Frackowiak, & Passingham, 1998; Oosterhof, Tipper, & Downing, 2013). Additionally, the Word×Stage interaction effect was significant in the left vPM (*F*_6,108_ = 2.567, *p *<* *.05), right vPM (*F*_6,108_ = 3.498, *p *<* *.01), left STS (*F*_6,108_ = 2.944, *p *<* *.05), and right STS (*F*_6,108_ = 2.367, *p *<* *.05) and marginal significant over the right aIPS (*F*_6,108_ = 2.158, *p *=* *.053), indicating that the word effect showed in the imitation stage only. That is, object words induced the strongest activity over these regions only in the imitation stage (Figure 4). To confirm this result, we also performed a one‐way ANOVA for the imitation stage and found a significant word effect over the right aIPS (*F*_3,54_ = 3.402, *p *<* *.05), left aIPS (*F*_3,54_ = 3.272, *p *<* *.05), right vPM (*F*_3,54_ = 5.663, *p *<* *.01), left vPM (*F*_3,54_ = 5.230, *p *<* *.01), and left STS (*F*_3,54_ = 8.095, *p *<* *.001), and a nearly significant effect over the right STS (*F*_3,54_ = 2.443, *p* = .074). Such a main effect of word type indicated the highest activation of the object word condition and a significantly or nearly significantly higher activity for the object word than the subject word condition over all six brain regions of interest, *p*s* *< .084 (see Figure 4).

## 4 DISCUSSION

In daily life, we are faced with all manner of action observation and imitation situations, which are accompanied by word cues (e.g., the body part, verbs, and objects) that may be task‐related or task‐irrelevant. This study serves as the first investigation of the mediating relationship between task‐irrelevant words and the brain activation during action observation and imitation. Our findings show that the word type modulates the neural activity in mirror neuron areas in the imitation stage, but not in the action observation stage. Specifically, we found a main effect of word type in the left precentral gyrus, inferior parietal lobule, right postcentral gyrus, and inferior frontal gyrus, which regions are nodes of the human MNS (Buccino, Vogt, et al., 2004; Cattaneo & Rizzolatti, 2009). In contrast to the subject word, verb, and checkerboard conditions, the hand‐related object words tended to be associated with increased activity in the left precentral gyrus, left inferior parietal lobule, right postcentral gyrus, and right inferior frontal gyrus. Considering that object words refer to the goal of actions, such enhancement in the object word condition provides further evidence that the MNS is associated with the encoding of the goal of action and high‐level cognitive process, such as inferring the intentions of others. The object of action is considered to be critical for understanding what the individual is doing (Ortigue, Sinigaglia, Rizzolatti, & Grafton, 2010; Ortigue, Thompson, Parasuraman, & Grafton, 2009). Our findings about the unique nature of the object word are therefore compatible with previous works that emphasized the goal understanding function of the MNS(Rizzolatti et al., 2014; Sperduti, Guionnet, Fossati, & Nadel, 2014). For instance, Biagi, Cioni, Fogassi, Guzzetta, and Tosetti (2010) examined anterior intraparietal cortex activity when participants observed complex object manipulation (e.g., inserting a key in a lock and turning it) actions and simple actions without specific intentions. The results showed significantly stronger anterior intraparietal cortex activity during the observation of complex actions with clear intentions. Another study recording neural firing in monkeys also showed that neurons in F5 (region inferior frontal gyrus) were sensitive to specific action intentions (Bonini et al., 2010), which is consistent with the object word enhancement effect observed in IFG. In addition, meta‐analysis have indicated that the left fusiform gyrus is commonly activated in single word reading task (Turkeltaub, Eden, Jones, & Zeffiro, 2002). However, we did not find significant activity in the left fusiform gyrus during word phase. One possibility is that our main task is to ask participants to imitate the action, which may weaken the processing of word so that to survive a threshold for statistical significance.

Although the observation stage immediately follows the word stage, we failed to find a significant word type effect during the observation stage. Action observation is relatively automatic according to a previous study (Chong, Williams, et al., 2008), which may account for the lack of the word type effect in the action observation stage. As a passive process, action observation will automatically activate the motor and premotor cortex (Buccino, Binkofski, & Riggio, 2004; Buccino et al., 2001; Fadiga, Fogassi, Pavesi, & Rizzolatti, 1995), which does not need the information of intention or goal. However, action imitation is an active process, which involves both how the action is going and the outcome or target of the action. Therefore, the ANOVA results for ROIs in our findings show that action observation is not affected by the previously presented words. Another explanation for the lack of the word type effect in the observation stage is that the relatively higher activation in these brain regions during the observation stage may weaken the difference of word type, see Figures 2 and 3. Another confounding factor is that the observation stage is always before the imitation stage in the present task. Given that the action video clip was played during the entire observation stage, participants may have had no time to associate the observed action and the previously presented word. However, in the imitation stage, it might cost participants less effort in processing the movement of the action but give them more time to generate and interpret movements.

It is worth noting that we observed the enhancement of activity for word conditions relative to the checkerboard condition over the ROI of the left MFG/precentral gyrus (PrecG) in both the word stage and the imitation stage. The MFG, as previous studies have indicated, is a well‐known key part of the Chinese word processing brain network (Liu et al., 2006; Siok, Perfetti, Jin, & Tan, 2004). For example, it has been proposed as an area involving in the integration of visual orthographic information with phonology in Chinese (Tan, Laird, Li, & Fox, 2005). Therefore, it is sensible that words evoked stronger brain activity over this brain region rather than other mirror neuron areas during the word stage. The word enhancement effect in the PrecG during the imitation stage, however, may reflect both the semantic‐action conflict and action inhibition. Given that words induced stronger responses within this area in the word stage, we believe that the words were processed, with the resulting perception inconsistent with the action in the imitation stage. Such a semantic‐action conflict may account for the increased activity in this area, as there is converging evidence on the role of this brain region in action inhibition, such as the inhibition of the imitation response tendency and the imitation of incongruent actions (Bien, Roebroeck, Goebel, & Sack, 2009; Brass, Derrfuss, & von Cramon, 2005; Brass, Zysset, & von Cramon, 2001).

Our findings also indicate an enhancement of IFG activity for object words, in contrast with other words or the control condition, in the imitation stage. It is well known that the IFG (BA44) is an important part of the language system and also the MNS. We consider that the activity in the IFG may be associated with action intention processing and the task‐irrelevant object words may induce more intention inferential processing, which has been suggested by previous studies. An fMRI study found that the right IFG responds differently to different intentional actions and proposed that the right IFG was associated with intention coding (Iacoboni et al., 2005b). Moreover, the inferior frontal gyrus was also demonstrated to be activated when observing the goals of hand–object interactions (Johnson‐Frey et al., 2003). Another fMRI study investigating brain activity during the observation of actions embedded or not embedded in contexts also confirmed the intention understanding function of the inferior frontal gyrus (Iacoboni et al., 2005b). Damage to the IFG has been shown to be associated with action understanding (e.g., gesture discrimination) deficits (Pazzaglia, Smania, Corato, & Aglioti, 2008). The role of the IFG in understanding action intention was confirmed by another study, which found that the activation in the IFG increased when the action intention is unusual (De Lange, Spronk, Willems, Toni, & Bekkering, 2008). In this study, IFG activity was a summation of typical mirror neuron activity and the coding of intention behind the action. In other words, action imitation following the observation recruits the classic MNS, including the right IFG. In addition, task‐irrelevant object words may initiate more intention inference processing. Therefore, the IFG activity was relatively stronger for object words than for the other three types of stimuli in the imitation stage.

Similar to the IFG, there is also evidence that the IPL serves as a neuronal substrate underlying the action outcome representation or intention understanding (Bonini et al., 2010; Fogassi et al., 2005; A. F. Hamilton & Grafton, 2008; A. F. D. Hamilton & Grafton, 2006). Strong evidence from Desmurget et al. (2009) suggested that electrical stimulation over the inferior parietal regions trigger a strong movement intention and desire. Accordingly, the observed higher signal change within the IPL for the object word condition may also be attributed to the intention inferential process. We thus propose that activity in both the IPL and the IFG reflect the intention understanding of actions, and our results show highest activity for object words in these two regions. Compelling previous studies have also shown that patients with brain lesions in the IFG or IPL have deficits in action understanding (Fazio et al., 2009).

As previously mentioned, the action understanding theory of mirror neurons was proposed by researchers as a solution for the problems in existing studies (Gallese, Gernsbacher, Heyes, Hickok, & Iacoboni, 2011; Hickok, 2009). The findings of this study may provide further support for the action understanding theory of the MNS. That is, as a part of the motor chain, the object words induced relatively higher activation in the IPL, IFG, premotor cortex, and STS. One possible explanation for why the object words induced stronger activity in the classic mirror neuron areas is that the task‐irrelevant object words evoked conflicts after the action observation stage and in response, participants increased their attention to finish their imitation task. One similar previous study showed that stimuli following multisensory conflicts induced enhanced attention, which was reflected by larger N1 and P2 responses to the following stimuli (Donohue, Todisco, & Woldorff, 2013). In this line of reasoning, task‐irrelevant words may elicit the conflict between the object and the action and make participants increase their brain response during imitation. Another possible explanation is that the object words lessen the adaptation effect over the MNS nodes. Specifically, repeating or execution of the observed action in the imitation stage showing an overall decreased activity in these brain regions (see Figures 3 and 4). However, for the object word condition, such an adaptation effect or suppression was decreased for greater intention representation, which recruits these brain regions.

The task potentially limited the interpretation of our results with regard to the word type effect on the MNS. Due to the observation–imitation task, the interval to imitation was always longer than that to observation. This suggested that our inference of word effect was only observed in the imitation stage, which was confounded with a temporal delay.It remains to be determined whether our findings would be the same if we control these factors during the task.

Taken together, our findings suggest that the MNS could be modulated by different action‐related words in the imitation stage. The object word enhanced brain activation in the imitation stage, which is consistent with the view that motor imitation is goal‐directed (Bekkering, Wohlschlager, & Gattis, 2000). That is, the action execution is implicitly modulated by the meaning of the object word, suggesting that the linguistic situation shapes action imitation (Garcia & Ibanez, 2016). Our results provide insight about the role of verbal instructions in skill teaching, such as how to give effective word or instructions in motor learning. It may be applicable to artificial motor learning that delivering goal‐directed linguistic instructions especially in context with actions and verbal information as well (Calinon, Guenter, & Billard, 2005; Erlhagen et al., 2006).

## CONFLICT OF INTEREST

None declared.
