# Supplementary figures and images for: Object words modulate the activity of the mirror neuron system during action imitation
Source: Brain Behav. 2017 Sep 26;7(11):e00840. doi: 10.1002/brb3.840 (PMC5698860; doi:10.1002/brb3.840)

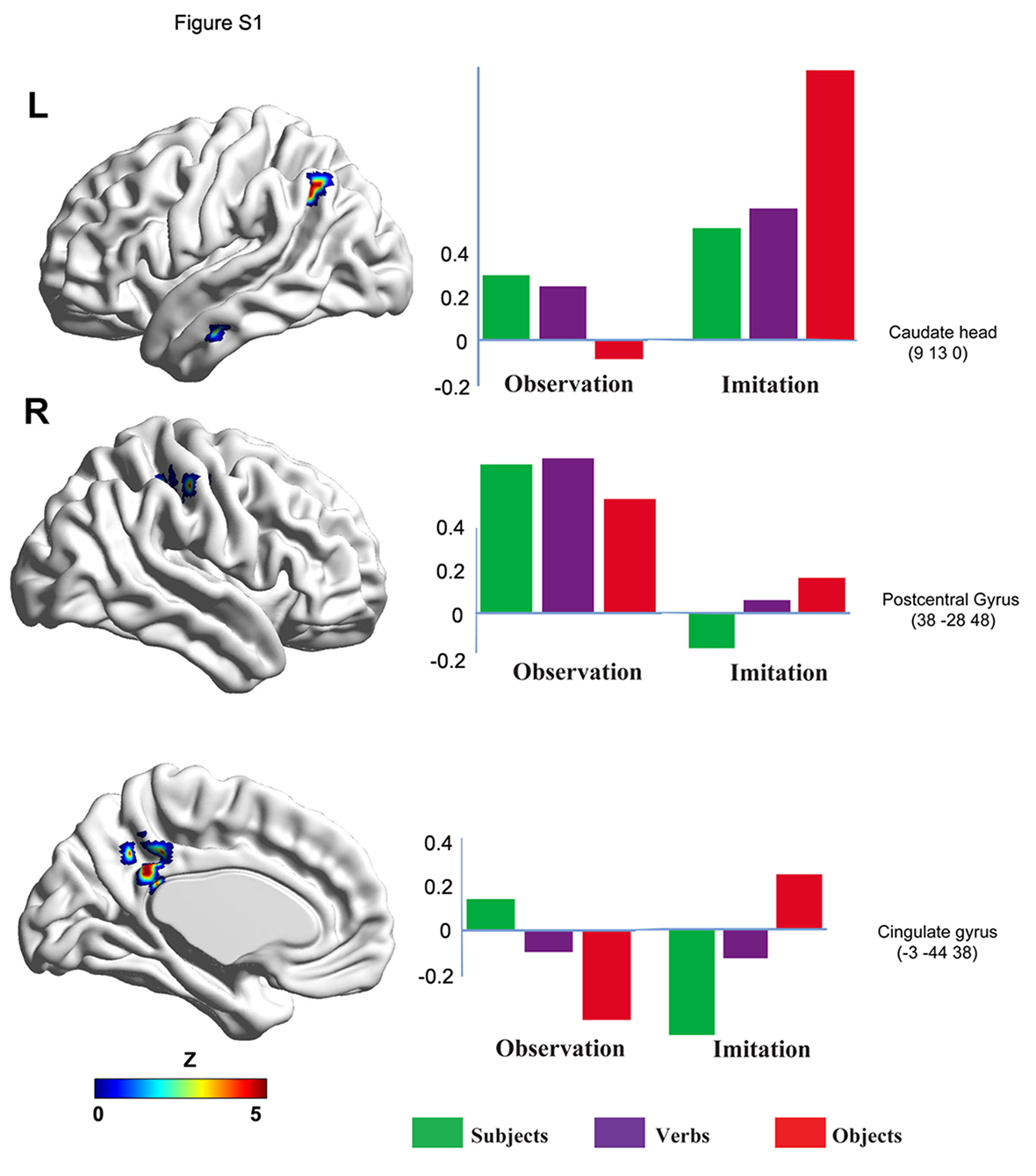

Supplement: Supplementary file 1 [file BRB3-7-e00840-s001.tif]
