# Supplementary material for: Object words modulate the activity of the mirror neuron system during action imitation
Source: Brain Behav. 2017 Sep 26;7(11):e00840. doi: 10.1002/brb3.840 (PMC5698860; doi:10.1002/brb3.840)
Supplement: Supplementary file 2 [file BRB3-7-e00840-s002.doc]

***Table S1. Coordinates and statistics for activation peaks in the interaction effect of 3WORD (subject, verb, and object) ×2 PHASE (observation and imitation) ANOVA***

|  | |  | **BA** | **x** | **y** | **z** | ***F*** | ***Z*** |
| --- | --- | --- | --- | --- | --- | --- | --- | --- |
| Postcentral gyrus | R | | 3 | 38 | -28 | 48 | 12.76 | 4.25 |
| Caudate head | R | | 40 | 9 | 13 | 0 | 10.73 | 3.86 |
| Inferior Parietal Lobe | L | |  | -47 | -59 | 43 | 10.62 | 3.84 |
| Cingulate Gyrus | L | | 31 | -3 | -44 | 38 | 9.61 | 3.63 |
| Precuneus | L | |  | -6 | -53 | 38 | 9.02 | 3.49 |
